# Detection of *Plasmodium falciparum* malaria by 16S rDNA testing in pericardial fluid from a blood antigen and microscopy negative patient

**DOI:** 10.1128/asmcr.00216-25

**Published:** 2026-07-23

**Authors:** Joseph J. Zeppa, Andrew K. Kapoor, Deborah Yamamura, Mark Loeb, Antoine Corbeil, Julianne V. Kus

**Affiliations:** 1 Department of Laboratory Medicine and Pathobiology, University of Toronto 233837, Toronto, Canada; 2 Division of Infectious Diseases, Department of Medicine, McMaster University 152997, Hamilton, Canada; 3 Division of General Internal Medicine, Department of Medicine, McMaster University 152997, Hamilton, Canada; 4 Microbiology Department, Hamilton Regional Laboratory Medicine Program, Hamilton Health Sciences and St. Joseph’s Healthcare 709550, Hamilton, Canada; 5 Department of Pathology and Molecular Medicine, McMaster University 153000, Hamilton, Canada; 6 Department of Health Research Methodology, Evidence, and Impact, McMaster University 3710, Hamilton, Canada; 7 Public Health Ontario 153300, Toronto, Canada; Pattern Bioscience, Austin, Texas, USA

**Keywords:** malaria, *Plasmodium falciparum*, 16S rDNA testing

## Abstract

**Background:**

Malaria is typically diagnosed using rapid antigen tests and microscopy. This case describes an unusual presentation of *Plasmodium falciparum*, initially detected via 16S rDNA testing of pericardial fluid identifying apicoplast DNA—a finding not previously reported in the literature.

**Case Summary:**

A septuagenarian Nigerian man visiting Canada presented with shortness of breath, tachycardia, tachypnea, anemia, and a pericardial effusion. Pericardiocentesis yielded serosanguineous fluid, and imaging revealed multifocal opacities. Blood and fluid cultures were negative for bacterial organisms. After briefly leaving the hospital against medical advice, he returned in respiratory distress and required intubation for acute respiratory distress syndrome. He received empiric antibiotics for rickettsial infection (due to positive serology) and presumed sepsis. Further workup, including pericardial fluid 16S rDNA PCR and sequencing, identified *P. falciparum* apicoplast DNA. This was confirmed by malaria antigen and *Plasmodium*-specific PCR testing of the fluid alongside PCR of whole blood, though microscopy and antigen tests remained negative. Hemozoin-like structures were also identifiable in the pericardial fluid. He was treated with a 3-day course of atovaquone-proguanil for malaria, with subsequent clinical improvement. He remains well on follow-up.

**Conclusion:**

This case underscores an uncommon presentation of *P. falciparum* and, though not a standard or recommended diagnostic tool for malaria, in this case, 16S rDNA analysis aided in the diagnosis. Clinicians should maintain malaria in the differential for patients from endemic regions. Multidisciplinary collaboration was essential in achieving the diagnosis.

## INTRODUCTION

Malaria remains a global concern, with ~282 million cases and 610,000 deaths estimated in 2024 (1). *Plasmodium falciparum* is responsible for severe forms of malaria and can present with a range of manifestations (cerebral malaria, anemia, renal failure, and acute respiratory distress syndrome) (2). Diagnosis often relies on antigen tests and microscopy (3). While molecular techniques have enhanced diagnostic sensitivity, their use is generally limited to specialized laboratories (3, 4).

16S rDNA PCR and sequence analysis is a powerful tool for prokaryotic pathogen identification, particularly in “culture-negative” infections (5, 6). This method targets conserved ribosomal RNA gene regions, allowing detection and identification based on sequence variation (7). Though not designed to detect eukaryotes, mitochondrion (8) or apicoplast (9) sequence identification can occur due to their prokaryotic origin (10).

This report describes an unexpected detection of *P. falciparum* infection in an afebrile Nigerian visiting Canada. Malaria was not initially suspected, but due to inconclusive microbiological results, 16S rDNA PCR was performed on pericardial fluid and identified *Plasmodium* apicoplast DNA—a finding not previously reported in the literature. This case underscores the importance of considering malaria after exposure to endemic regions despite uncommon manifestations and highlights the role of molecular diagnostics and multidisciplinary collaboration in achieving a definitive diagnosis.

## CASE PRESENTATION

**Fig 1:**
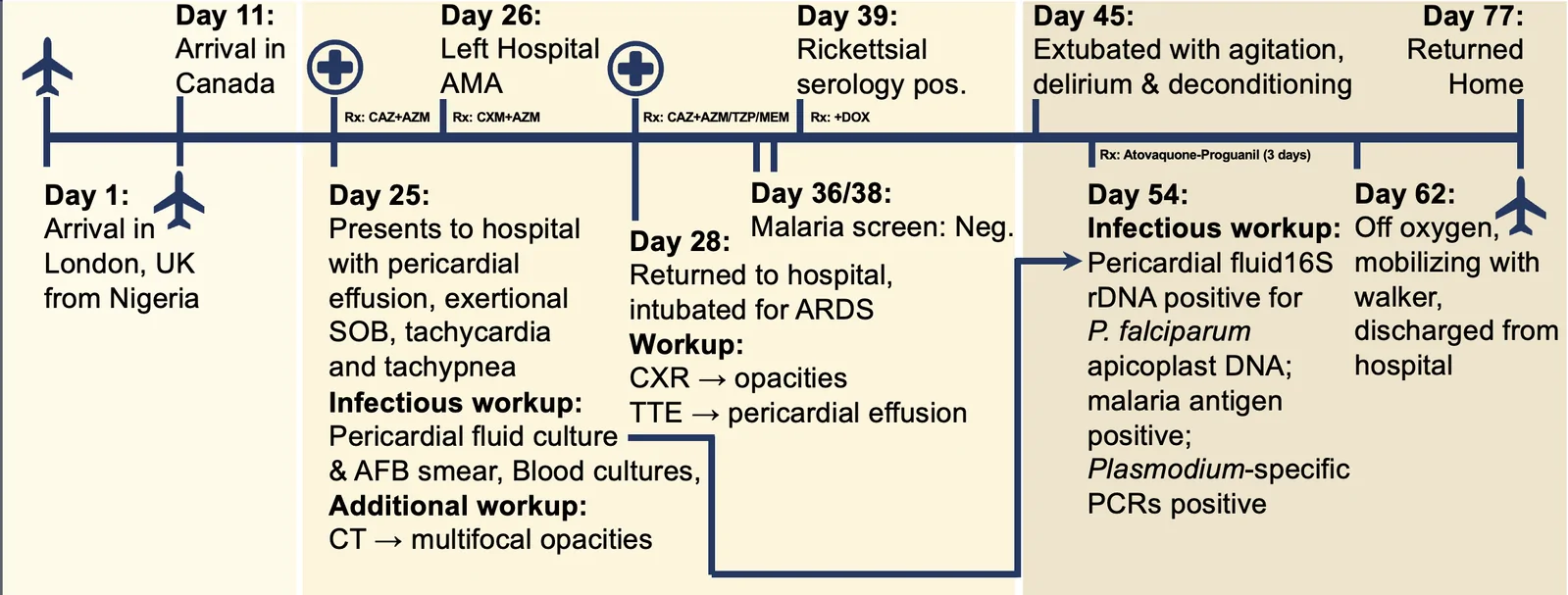
Timeline of important events in the presented case. SOB, shortness of breath; AFB, acid-fast bacilli; CT, computed tomography; ARDS, acute respiratory distress syndrome; CXR, chest X-ray; TTE, transthoracic echocardiogram; CRO, ceftriaxone; AZM, azithromycin; CXM, cefuroxime; TZP, piperacillin-tazobactam; MEM, meropenem; DOX, doxycycline.

A septuagenarian Nigerian man with no significant medical history was visiting relatives in Canada and was admitted to a tertiary-care facility 25 days into their trip with afebrile tachycardia, tachypnea, and pericardial effusion (PE) without tamponade (Fig. 1). Pericardiocentesis fluid (240 cc) was sent for microscopy and culture (bacterial and mycobacterial), which were negative. Computed tomography showed multifocal right lower lobe ground-glass opacities. He was started on ceftriaxone and azithromycin for possible pneumonia. One day later (day 26), he left the hospital against medical advice due to uninsured status but was given cefuroxime and azithromycin for home use.

On day 28, he returned to the emergency department with respiratory distress requiring intubation. Chest X-ray (CXR) displayed multifocal opacities, and transthoracic echocardiogram showed a PE with fibrinous material and no tamponade. Ceftriaxone and azithromycin were restarted. On day 32, bronchoscopy demonstrated mucus in both primary bronchi, with X-rays showing complete opacification of the left lung. On day 34, the patient became febrile (38.8°C) and was given piperacillin-tazobactam, which was changed to meropenem after a positive extended-spectrum beta-lactamase colonization testing. Whole blood thick and thin smear microscopy and antigen testing (BinaxNOW Malaria, Abbott, Scarborough, Maine, US) were negative for malaria on days 36 and 38. Doxycycline was added on day 39 after a positive rickettsial serology result (Focus Diagnostics Indirect Immunofluorescent Antibody IgG, Cypress, California, US) with spotted fever group (SFG) titer 1:128 and typhus group (TG) titer 1:128. CXR on day 42 demonstrated bilateral mixed interstitial and airspace opacities with reticulonodular configurations, but no worsening consolidations, effusions, or pneumothorax. On day 44, HIV and syphilis results were negative, and the next day, the patient was extubated. Recovery was complicated by agitation, delirium, and decompensation.

**Fig 2:**
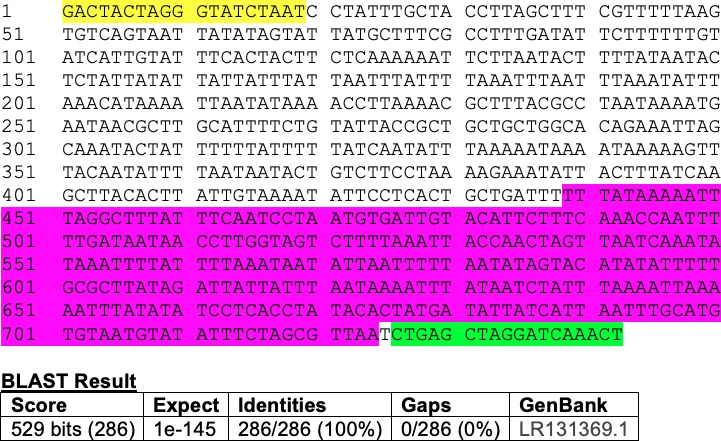
Alignment of sequencing results from 16S rDNA gene testing to *Plasmodium falciparum* apicoplast organelle sequence. Pericardial fluid from the patient underwent 16S rDNA gene PCR followed by Sanger sequencing. Top: The primers used in this study (8FLP [5’–AGTTTGATCCTGGCTCAG–3’; reverse complement in green] and 806R [5’–GGACTACCAGGGTATCTAAT–3’; the last 19 nucleotides highlighted in yellow]) mapped onto the *P. falciparum* apicoplast organelle DNA (GenBank ID: LR131369.1). When the sample was run, the PCR amplified a band ~740 bp in length. Sanger sequencing of that band revealed a string of 286 uninterrupted base pairs (fuchsia), that when entered into the National Institutes of Health (NIH) National Center for Biotechnology Information (NCBI) Basic Local Alignment Search Tool (BLAST), returned an exact match to *P. falciparum* apicoplast organelle DNA. The BLAST search parameters were as follows: BLAST nucleotide (blastn), standard database, models (XM/XP), and uncultured/environmental sample sequences excluded with optimization for highly similar sequences (megablast). Bottom: BLAST results from this study.

**Fig 3:**
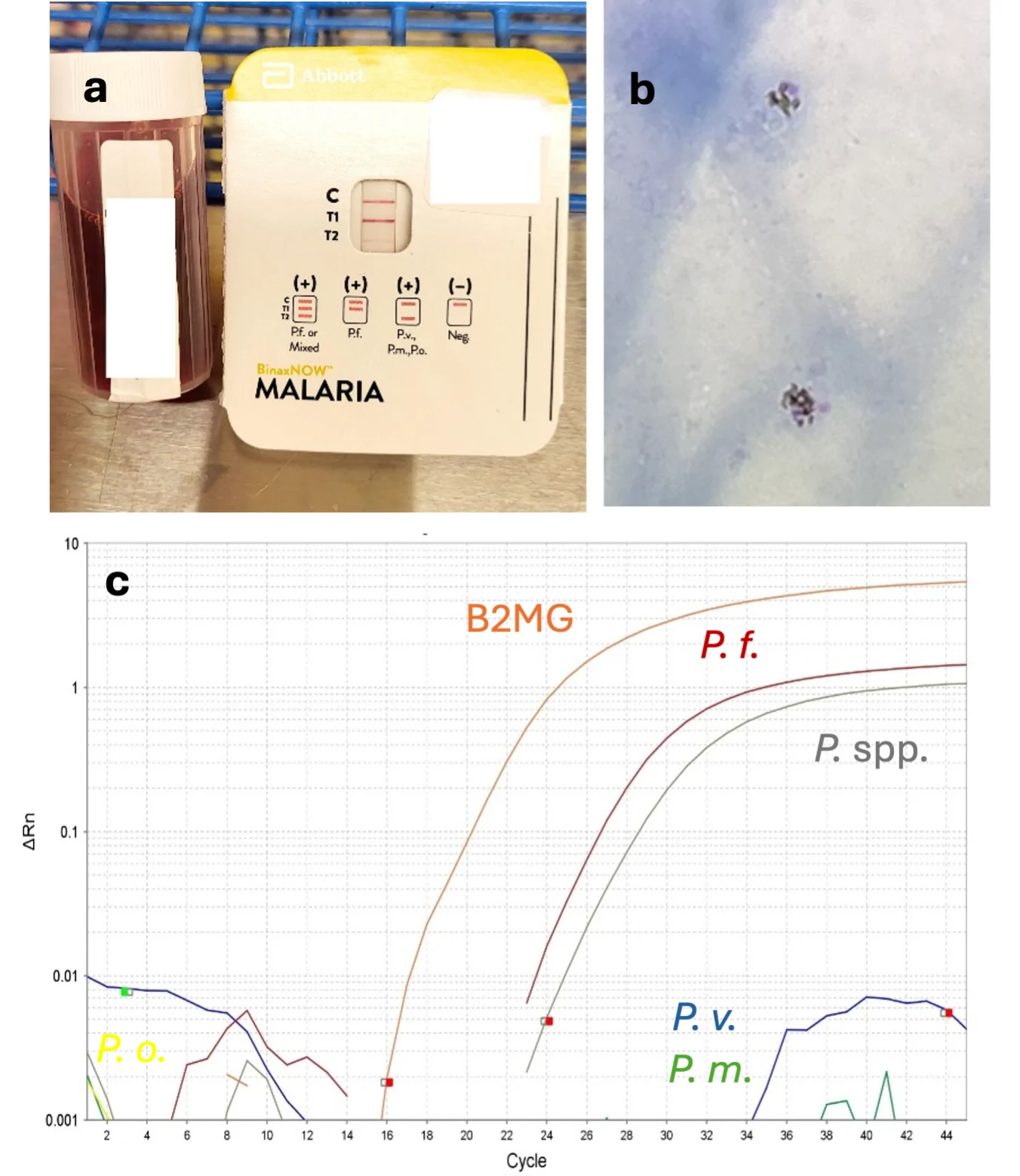
Patient malaria testing results from day 54. (**a**) Pericardial fluid sample (left) with corresponding antigen test result (right) tested directly from the fluid. Patient label identifiers have been redacted. (**b**) Giemsa microscopy (1,000× magnification) from the pericardial fluid demonstrating multiple refractile pigmented dark brown structures resembling hemozoin. No typical malarial organisms were identifiable. (**c**) Multiplex real-time PCR logarithmic amplification plot demonstrating detection of *Plasmodium falciparum* (*P. f*.) and *Plasmodium* species (*P*. spp.) 18S rRNA gene targets. The human beta-2-microglobulin (B2MG) internal control target was also amplified, while no detection was observed for *P. vivax* (*P. v*.), *P. ovale* (*P. o*.), or *P. malariae* (*P. m*.).

On day 54, 16S rDNA PCR and sequencing on the pericardial fluid (from day 26) was positive for *P. falciparum* apicoplast DNA based on high similarity sequence alignment in the National Institutes of Health National Center for Biotechnology Information Basic Local Alignment Search Tool (Fig. 2) (11, 12). The clinical team was informed of the unusual result, and the test was not validated for parasite detection. Additional malaria-specific testing was performed on the pericardial fluid for result confirmation. BinaxNOW Malaria HRP-2 and aldolase antigens (Fig. 3a) and two different 18S rDNA PCR targets (Fig. 3b) were positive: *Plasmodium* genus 18S (Ct28) and *P. falciparum* 18S (Ct26) (13–15). Pericardial fluid microscopy revealed dark brownish refractile structures resembling hemozoin (Fig. 3c). Whole blood collected on day 54 was microscopy and antigen negative but PCR indeterminate (*Plasmodium* genus 18S Ct43 and *P. falciparum* 18S Ct41). Repeat rickettsial serology on day 59 was borderline positive (SFG titer 1:64; TG titer 1:64).

After the 16S result, the patient was treated for malaria (3-day course of atovaquone-proguanil) and improved clinically. By day 62, they were off supplemental oxygen, mobilizing with a walker, and discharged home.

## DISCUSSION

This case report highlights an unusual malaria presentation of PE without typical symptoms (fever paroxysms, fatigue, chills, headache, abdominal pain, and diarrhea) (2). Importantly, malaria testing was negative after the first in-hospital episode of fever 11 days after admission.

Cardiac complications are not often highlighted in malaria literature. A review by Gupta et al. outlines reported cardiovascular symptoms including myocarditis and pericarditis (16), with reported data showing that pericardial effusion may occur in up to 9.7% of cases (17–19).

Malaria diagnostics have limitations. Microscopy has relatively high clinical sensitivity (85%–93%) (20, 21), but user variation and other factors (periodicity and sequestration) can cause false negatives (22–24). Antigen tests have varying clinical sensitivities depending on species and false negatives from parasitemia extremes or genetic mutations (4, 25, 26). Sensitivity of conventional tests in semi-immune individuals with subclinical infections is less optimal (27, 28). In this circumstance, access to more sensitive diagnostic options (PCR or loop-mediated isothermal amplification) (29, 30), as supported by Canadian guidelines, may have detected malaria sooner (4). Social factors also impacted management, with the patient leaving due to a lack of insurance only to return with more severe disease.

This case’s most unique feature was the detection of *P. falciparum* apicoplast DNA by a bacteria-specific detection method. We were unable to find another report describing a similar scenario for malaria; however, a case of cerebral toxoplasmosis has been diagnosed via incidental 16S PCR (9). Although unexpected, our finding is compatible with the prokaryotic origins of apicoplasts and their harboring 16S ribosomal sequences (10, 31).

It should be noted that our 16S rDNA sequencing was not validated to detect parasite DNA. Although not validated for this specimen, the positive *Plasmodium* antigen and PCR tests on the pericardial fluid corroborated this unexpected result. The negative pericardial microscopy was probably due to prolonged blood storage, but suspect hemozoin structures were noted. Unfortunately, earlier blood samples were unavailable for retrospective testing. Overall, malarial infection likely drove the clinical presentation with improvement after malaria-specific treatment. It is important to note that atovaquone-proguanil is not the preferred treatment for acutely presenting severe malaria (4). In this case, once malaria was detected, there was no evidence of parasitemia, no severe disease features, and the patient was stable. These factors and a clinical risk assessment supported this therapeutic approach. Interestingly, the patient had no recollection of past malarial infections, testing, or treatment. They are, however, from Nigeria, which has the highest prevalence of malaria globally (1). Although unconfirmed, semi-immunity from prior exposure may explain the atypical presentation and the negative initial test results. Our patient was also borderline *Rickettsia* seropositive, and PE is rarely reported in rickettsial infection (32, 33), but the patient remained unwell after doxycycline. The low-level titers, cross-reactive patterns for both SFG and TG, and the lack of increasing titers over time may suggest non-specific seroreactivity or unrelated prior exposure.

Subclinical and submicroscopic malaria infections are more common among those from endemic areas, which may contribute to delayed diagnosis and ongoing transmission risk (34, 35). Mixed-species infections are also increasingly recognized through molecular diagnostics and may complicate treatment by masking resistant or relapsing species (36–38). Although our patient was not subclinical, and mixed infection was unlikely due to our highly sensitive and specific PCR detecting only *P. falciparum* malaria, these are important considerations during clinically complex scenarios in those from endemic countries.

A major take-home message is that malaria should remain on the differential for patients from endemic regions, even with negative routine tests. In complex scenarios, a multidisciplinary team of infectious disease physicians, microbiologists, and tropical disease specialists may be helpful to address malaria risk and unexpected laboratory findings. It bears repeating that 16S rDNA PCR should not be used as first-line testing when parasitic infections are suspected. Unanticipated results should be interpreted cautiously but never ignored. The utility of broad-scale diagnostic methodologies in complex clinical scenarios is increasingly recognized, such as with metagenomics (39), cell-free DNA analysis (40), and multiplex arrays (41).

In conclusion, this publication outlines a complex scenario whereby *P. falciparum* malaria was detected by 16S rDNA sequencing. This case highlights non-traditional diagnostic methods yielding critical insights. In these scenarios, diagnostic flexibility and interdisciplinary communication are paramount to providing optimal patient care.

## Data Availability

Data are available upon request.

## References

[1] WHO. 2025. World malaria report. https://www.who.int/teams/global-malaria-programme/reports/world-malaria-report-2025.

[2] Daily JP, Parikh S. 2025. Malaria. N Engl J Med 392:1320–1333. doi:10.1056/NEJMra2405313 40174226 PMC12331251

[3] Fitri LE, Widaningrum T, Endharti AT, Prabowo MH, Winaris N, Nugraha RYB. 2022. Malaria diagnostic update: from conventional to advanced method. J Clin Lab Anal 36:e24314. doi:10.1002/jcla.24314 35247002 PMC8993657

[4] Canada PHA. 2019. Canadian recommendations for the prevention and treatment of malaria: an advisory committee statement (ACS). Committee to Advise on Tropical Medicine and Travel (CATMAT)

[5] Janda JM, Abbott SL. 2007. 16S rRNA gene sequencing for bacterial identification in the diagnostic laboratory: pluses, perils, and pitfalls. J Clin Microbiol 45:2761–2764. doi:10.1128/JCM.01228-07 17626177 PMC2045242

[6] Church DL, Cerutti L, Gürtler A, Griener T, Zelazny A, Emler S. 2020. Performance and application of 16S rRNA gene cycle sequencing for routine identification of bacteria in the clinical microbiology laboratory. Clin Microbiol Rev 33:1–74. doi:10.1128/CMR.00053-19 PMC7484979 32907806

[7] Petti CA, Bosshard PP, Brandt ME, Clarridge JE, Foxall P, Furtado MR, Pace N, Procop G. 2008. Interpretive criteria for identification of bacteria and fungi by DNA target sequencing: approved guideline. Clinical and Laboratory Standards Institute

[8] Walker SP, Barrett M, Hogan G, Flores Bueso Y, Claesson MJ, Tangney M. 2020. Non-specific amplification of human DNA is a major challenge for 16S rRNA gene sequence analysis. Sci Rep 10:16356. doi:10.1038/s41598-020-73403-7 33004967 PMC7529756

[9] Kruse AYC, Kvich L, Eickhardt S, Omland LH, Bjarnsholt T, Moser C. 2015. Unexpected diagnosis of cerebral toxoplasmosis by 16S and D2 large-subunit ribosomal DNA PCR and sequencing. J Clin Microbiol 53:1983–1985. doi:10.1128/JCM.03404-14 25854484 PMC4432055

[10] Biddau M, Sheiner L. 2019. Targeting the apicoplast in malaria. Biochem Soc Trans 47:973–983. doi:10.1042/BST20170563 31383817

[11] Altschul SF, Gish W, Miller W, Myers EW, Lipman DJ. 1990. Basic local alignment search tool. J Mol Biol 215:403–410. doi:10.1016/S0022-2836(05)80360-2 2231712

[12] Camacho C, Coulouris G, Avagyan V, Ma N, Papadopoulos J, Bealer K, Madden TL. 2009. BLAST+: architecture and applications. BMC Bioinformatics 10:421. doi:10.1186/1471-2105-10-421 20003500 PMC2803857

[13] Rougemont M, Van Saanen M, Sahli R, Hinrikson HP, Bille J, Jaton K. 2004. Detection of four *Plasmodium* species in blood from humans by 18S rRNA gene subunit-based and species-specific real-time PCR assays. J Clin Microbiol 42:5636–5643. doi:10.1128/JCM.42.12.5636-5643.2004 15583293 PMC535226

[14] Shokoples SE, Ndao M, Kowalewska-Grochowska K, Yanow SK. 2009. Multiplexed real-time PCR assay for discrimination of *Plasmodium* species with improved sensitivity for mixed infections. J Clin Microbiol 47:975–980. doi:10.1128/JCM.01858-08 19244467 PMC2668309

[15] Phuong M, Lau R, Ralevski F, Boggild AK. 2014. Sequence-based optimization of a quantitative real-time PCR assay for detection of *Plasmodium ovale* and *Plasmodium malariae*. J Clin Microbiol 52:1068–1073. doi:10.1128/JCM.03477-13 24430459 PMC3993485

[16] Gupta S, Gazendam N, Farina JM, Saldarriaga C, Mendoza I, López-Santi R, Pérez GE, Martínez-Sellés M, Baranchuk A. 2021. Malaria and the heart: JACC state-of-the-art review. J Am Coll Cardiol 77:1110–1121. doi:10.1016/j.jacc.2020.12.042 33632486

[17] Franzen D, Curtius JM, Heitz W, Höpp HW, Diehl V, Hilger HH. 1992. Cardiac involvement during and after malaria. Clin Investig 70:670–673. doi:10.1007/BF00180283 1392443

[18] Ray HN, Doshi D, Rajan A, Singh AK, Singh SB, Das MK. 2017. Cardiovascular involvement in severe malaria: a prospective study in Ranchi, Jharkhand. J Vector Borne Dis 54:177–182. doi:10.4103/0972-9062.211700 28748840

[19] Kaagaard MD, Matos LO, Holm AE, Gomes LC, Wegener A, Lima KO, Vieira IVM, de Souza RM, Marinho CRF, Hviid L, Vestergaard LS, Dominguez H, Biering-Sørensen T, Silvestre OM, Brainin P. 2022. Frequency of electrocardiographic alterations and pericardial effusion in patients with uncomplicated malaria. Am J Cardiol 165:116–123. doi:10.1016/j.amjcard.2021.11.009 34906368

[20] Stauffer WM, Cartwright CP, Olson DA, Juni BA, Taylor CM, Bowers SH, Hanson KL, Rosenblatt JE, Boulware DR. 2009. Diagnostic performance of rapid diagnostic tests versus blood smears for malaria in US clinical practice. Clin Infect Dis 49:908–913. doi:10.1086/605436 19686072 PMC2912215

[21] Johnston SP, Pieniazek NJ, Xayavong MV, Slemenda SB, Wilkins PP, da Silva AJ. 2006. PCR as a confirmatory technique for laboratory diagnosis of malaria. J Clin Microbiol 44:1087–1089. doi:10.1128/JCM.44.3.1087-1089.2006 16517900 PMC1393165

[22] Manser M, Olufsen C, Andrews N, Chiodini PL. 2013. Estimating the parasitaemia of *Plasmodium falciparum*: experience from a national EQA scheme. Malar J 12:1–7. doi:10.1186/1475-2875-12-428 24261625 PMC4222811

[23] Beaudry JT, Fairhurst RM. 2011. Microvascular sequestration of *Plasmodium falciparum*. Blood 117:6410. doi:10.1182/blood-2010-09-305102 21823261 PMC3123013

[24] Calderaro A, Montecchini S, Buttrini M, Piccolo G, Rossi S, Arcangeletti MC, Farina B, De Conto F, Chezzi C. 2021. Malaria diagnosis in non-endemic settings: the European experience in the last 22 years. Microorganisms 9:1–25. doi:10.3390/microorganisms9112265 PMC8620059 34835391

[25] Maltha J, Gillet P, Jacobs J. 2013. Malaria rapid diagnostic tests in endemic settings. Clin Microbiol Infect 19:399–407. doi:10.1111/1469-0691.12151 23438048

[26] Farcas GA, Zhong KJY, Lovegrove FE, Graham CM, Kain KC. 2003. Evaluation of the Binax NOW ICT test versus polymerase chain reaction and microscopy for the detection of malaria in returned travelers. Am J Trop Med Hyg 69:589–592. doi:10.4269/ajtmh.2003.69.6.0690589 14740873

[27] Ojeniyi FD, Ayoola AO, Ibitoye O, Opaleye OO, Olowe OA, Ehigie LO, Thomas BN, Ojurongbe O. 2025. Performance and challenges of malaria rapid diagnostic tests in endemic regions of Africa. Sci Rep 15:43663. doi:10.1038/s41598-025-97259-x 41381696 PMC12701009

[28] Ranadive N, Kunene S, Darteh S, Ntshalintshali N, Nhlabathi N, Dlamini N, Chitundu S, Saini M, Murphy M, Soble A, Schwartz A, Greenhouse B, Hsiang MS. 2017. Limitations of rapid diagnostic testing in patients with suspected malaria: a diagnostic accuracy evaluation from Swaziland, a low-endemicity country aiming for malaria elimination. Clin Infect Dis 64:1221–1227. doi:10.1093/cid/cix131 28369268 PMC5399938

[29] Rypien C, Chow B, Chan WW, Church DL, Pillai DR. 2017. Detection of *Plasmodium* infection by the illumigene malaria assay compared to reference microscopy and real-time PCR. J Clin Microbiol 55:3037–3045. doi:10.1128/JCM.00806-17 28768730 PMC5625390

[30] Cheaveau J, Nguyen H, Chow B, Marasinghe D, Mohon AN, Yuan H, Viana G, van Schalkwyk D, Church D, Chan W, Pillai DR. 2018. Clinical validation of a commercial LAMP test for ruling out malaria in returning travelers: a prospective diagnostic trial. Open Forum Infect Dis 5:ofy260. doi:10.1093/ofid/ofy260 30465012 PMC6239078

[31] Wilson (Iain) RJM, Denny PW, Preiser PR, Rangachari K, Roberts K, Roy A, Whyte A, Strath M, Moore DJ, Moore PW, Williamson DH. 1996. Complete gene map of the plastid-like DNA of the malaria parasite *Plasmodium falciparum*. J Mol Biol 261:155–172. doi:10.1006/jmbi.1996.0449 8757284

[32] Blanton LS. 2019. The rickettsioses: a practical update. Infect Dis Clin North Am 33:213–229. doi:10.1016/j.idc.2018.10.010 30712763 PMC6364315

[33] Po T-L, Huang C-H, Lin C-H, Hung H-F. 2024. Diagnosis of a rare *Rickettsia felis* infection complicated with unusual pericardial effusion and cardiac tamponade using an mNGS test. Case Rep Infect Dis 2024:8877876. doi:10.1155/2024/8877876 39165786 PMC11335410

[34] Ranjha R, Gupta A, Singh K, Sikka R, Anvikar AR, Gupta H, Bharti PK. 2026. Mechanistic insights into subclinical *Plasmodium* infections: unveiling the silent drivers of malaria transmission. iScience 29:115979. doi:10.1016/j.isci.2026.115979 42181238 PMC13197720

[35] Whittaker C, Slater H, Nash R, Bousema T, Drakeley C, Ghani AC, Okell LC. 2021. Global patterns of submicroscopic *Plasmodium falciparum* malaria infection: insights from a systematic review and meta-analysis of population surveys. Lancet Microbe 2:e366–e374. doi:10.1016/S2666-5247(21)00055-0 34382027 PMC8332195

[36] Kotepui M, Kotepui KU, De Jesus Milanez G, Masangkay FR. 2020. *Plasmodium* spp. mixed infection leading to severe malaria: a systematic review and meta-analysis. Sci Rep 10:11068. doi:10.1038/s41598-020-68082-3 32632180 PMC7338391

[37] Mayxay M, Pukrittayakamee S, Newton PN, White NJ. 2004. Mixed-species malaria infections in humans. Trends Parasitol 20:233–240. doi:10.1016/j.pt.2004.03.006 15105024

[38] Zimmerman PA, Mehlotra RK, Kasehagen LJ, Kazura JW. 2004. Why do we need to know more about mixed *Plasmodium* species infections in humans? Trends Parasitol 20:440–447. doi:10.1016/j.pt.2004.07.004 15324735 PMC3728821

[39] Chiu CY, Miller SA. 2019. Clinical metagenomics. Nat Rev Genet 20:341–355. doi:10.1038/s41576-019-0113-7 30918369 PMC6858796

[40] Blauwkamp TA, Thair S, Rosen MJ, Blair L, Lindner MS, Vilfan ID, Kawli T, Christians FC, Venkatasubrahmanyam S, Wall GD, Cheung A, Rogers ZN, Meshulam-Simon G, Huijse L, Balakrishnan S, Quinn JV, Hollemon D, Hong DK, Vaughn ML, Kertesz M, Bercovici S, Wilber JC, Yang S. 2019. Analytical and clinical validation of a microbial cell-free DNA sequencing test for infectious disease. Nat Microbiol 4:663–674. doi:10.1038/s41564-018-0349-6 30742071

[41] Lewinski MA, Alby K, Babady NE, Butler-Wu SM, Bard JD, Greninger AL, Hanson K, Naccache SN, Newton D, Temple-Smolkin RL, Nolte F. 2023. Exploring the utility of multiplex infectious disease panel testing for diagnosis of infection in different body sites. A joint report of the association for molecular pathology, American Society for Microbiology, Infectious Diseases Society of America, and Pan American Society for Clinical Virology. J Mol Diagnostics 25:857–875. doi:10.1016/j.jmoldx.2023.08.005 PMC11702286 37757952

